# Item

**Published:** 1897-07

**Authors:** 


### Item.

THE dispensary abuse has received investigation at the hands of the Medical Society of the County of New York, which, in October last, appointed the following committee on the subject: J. H. Burtenshaw, chairman, A. B. Ball, Hermann J. Boldt, E. S. Bullock, Henry D. Chapin, Carter S. Cole, Alexander Hadden, William M. Polk, W. Washburn, W. H. Weston and F. H. Wiggin. The result of its deliberations was a carefully prepared bill, tending to correct the evils, and which passed both houses of the last legislature without opposition. It failed, however, to receive the governor's approval, hence the work must be done over again. It is only a matter of delay, not of defeat.

